# Genetic Effects on Transcriptome Profiles in Colon Epithelium Provide Functional Insights for Genetic Risk Loci

**DOI:** 10.1016/j.jcmgh.2021.02.003

**Published:** 2021-02-16

**Authors:** Virginia Díez-Obrero, Christopher H. Dampier, Ferran Moratalla-Navarro, Matthew Devall, Sarah J. Plummer, Anna Díez-Villanueva, Ulrike Peters, Stephanie Bien, Jeroen R. Huyghe, Anshul Kundaje, Gemma Ibáñez-Sanz, Elisabeth Guinó, Mireia Obón-Santacana, Robert Carreras-Torres, Graham Casey, Víctor Moreno

**Affiliations:** 1 Oncology Data Analytics Program, Catalan Institute of Oncology, L’Hospitalet de Llobregat, Barcelona, Spain; 2 Colorectal Cancer Group, Molecular Mechanisms and Experimental Therapy in Oncology (ONCOBELL) Program, Bellvitge Biomedical Research Institute, Spain; 3 Consortium for Biomedical Research in Epidemiology and Public Health, Madrid, Spain; 4 Department of Clinical Sciences, Faculty of Medicine, University of Barcelona, Barcelona, Spain; 5 Center for Public Health Genomics, University of Virginia, Charlottesville, Virginia; 6 Department of Public Health Sciences, University of Virginia, Charlottesville, Virginia; 7 Department of Surgery, University of Virginia, Charlottesville, Virginia; 8 Epidemiology Department, University of Washington, Seattle, Washington; 9 Public Health Sciences Division, Fred Hutchinson Cancer Research Center, Seattle, Washington; 10 Department of Genetics, Stanford University, Stanford, California; 11 Gastroenterology Department, Bellvitge University Hospital, L’Hospitalet de Llobregat, Barcelona, Spain

**Keywords:** Gene Expression, Alternative Splicing, QTLs, Colon, AS, alternative splicing, BarcUVa-Seq, University of Barcelona and University of Virginia genotyping and RNA sequencing project, CoTrEx, colon transcriptome explorer, CRC, colorectal cancer, eGene, eQTL gene, eQTL, expression quantitative trait locus, eSNP, eQTL SNP, FDR, false-discovery rate, FWER, family-wise error rate, GTEx, Genotype-Tissue Expression project, GWAS, genome-wide association study, LD, linkage disequilibrium, MAF, minor allele frequency, PSI, percent splicing index, RBP, RNA-binding proteins, RNA-Seq, RNA sequencing, sGene, sQTL gene, SNP, single-nucleotide polymorphism, sSNP, sQTL SNP, sQTL, splicing quantitative trait locus, TSS, transcription start site

## Abstract

**Background & Aims:**

The association of genetic variation with tissue-specific gene expression and alternative splicing guides functional characterization of complex trait-associated loci and may suggest novel genes implicated in disease. Here, our aims were as follows: (1) to generate reference profiles of colon mucosa gene expression and alternative splicing and compare them across colon subsites (ascending, transverse, and descending), (2) to identify expression and splicing quantitative trait loci (QTLs), (3) to find traits for which identified QTLs contribute to single-nucleotide polymorphism (SNP)-based heritability, (4) to propose candidate effector genes, and (5) to provide a web-based visualization resource.

**Methods:**

We collected colonic mucosal biopsy specimens from 485 healthy adults and performed bulk RNA sequencing. We performed genome-wide SNP genotyping from blood leukocytes. Statistical approaches and bioinformatics software were used for QTL identification and downstream analyses.

**Results:**

We provided a complete quantification of gene expression and alternative splicing across colon subsites and described their differences. We identified thousands of expression and splicing QTLs and defined their enrichment at genome-wide regulatory regions. We found that part of the SNP-based heritability of diseases affecting colon tissue, such as colorectal cancer and inflammatory bowel disease, but also of diseases affecting other tissues, such as psychiatric conditions, can be explained by the identified QTLs. We provided candidate effector genes for multiple phenotypes. Finally, we provided the Colon Transcriptome Explorer web application.

**Conclusions:**

We provide a large characterization of gene expression and splicing across colon subsites. Our findings provide greater etiologic insight into complex traits and diseases influenced by transcriptomic changes in colon tissue.

## Graphical abstract

**Figure:**
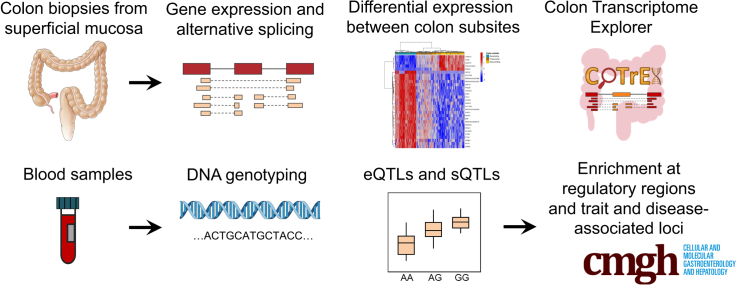

**Summary**

We profiled gene expression and alternative splicing of non-neoplastic colon from biopsy specimens from 445 healthy individuals. We showed that single-nucleotide polymorphisms associated with these profiles are enriched in disease-associated loci, including colorectal cancer and inflammatory bowel disease.

Transcriptome-wide gene expression profiles of normal colon tissue have been assessed in population-based studies, using data sets with a range of different characteristics, including variable colon anatomic subsites, collection methods, sample sizes, sequencing technologies, and data processing methods.^1^, ^2^, ^3^, ^4^, ^5^, ^6^, ^7^, ^8^ A large public transcriptome data set for non-neoplastic colon tissue from the Genotype-Tissue Expression (GTEx) project included samples collected from the transverse and sigmoid colon of post-mortem subjects and included both mucosa and muscularis propria.^8^ In most studies, the transcriptome is assessed in terms of gene expression, however, a comprehensive characterization of alternative splicing (AS) has not been performed in normal colon epithelial tissue derived from living individuals.

AS is a post-transcriptional regulatory mechanism by which multiple messenger RNA transcripts are produced from a single locus, enabling enlargement of cellular functions.^9^ More than 90% of human genes have the potential to undergo AS.^10^ Common AS patterns include exon skipping, alternative 5’ and 3’ splice sites, mutually exclusive exons, intron retention, and alternative first or last exons.^11^ Based on these predefined patterns and transcript expression levels, different AS events and their relative abundances can be identified for a given gene.^12^ In addition, by measuring alternative excision of introns, novel and more complex alternative splicing events can be identified.^13^ AS has been assessed in multiple tissue types across several large cohorts, including healthy^8^ and pathologic tissues,^14^, ^15^, ^16^ allowing the association of particular AS events with phenotypes such as age^17^ and cancer type.^14^, ^15^, ^16^ In colon tissue, AS events have been measured in adenocarcinomas and paired adjacent normal tissue and have been associated with colorectal cancer (CRC) anatomic location^18^ and prognosis.^18^, ^19^, ^20^

Single-nucleotide polymorphisms (SNPs) have been associated with gene expression (ie, expression quantitative trait loci [eQTLs]) and AS (sQTLs), and increasingly are identified in studies of both normal^8^^,^^21^, ^22^, ^23^, ^24^, ^25^ and malignant tissues.^26^ Such associations can indicate the functional effects of SNPs at genetic risk loci, help prioritize SNPs and genes for functional assays, serve as prognostic biomarkers, and suggest disease mechanisms.^10^^,^^26^^,^^27^ In the case of normal colon tissue, eQTL data sets have been generated,^1^, ^2^, ^3^, ^4^, ^5^, ^6^, ^7^, ^8^ but there is no information about sQTLs derived from living individuals.

In this study, we analyzed a novel RNA sequencing (RNA-Seq) data set of normal colon tissue biopsy specimens including colon anatomic subsites not investigated previously (ascending, transverse, and descending). Our data set is representative of the transcriptome of colon epithelial cells of living subjects because all biopsy specimens were collected from mucosa at colonoscopy. This characteristic makes it optimal for investigating the normal physiology across the colon, and it is relevant not only for studying the etiologic aspects of diseases affecting this tissue, such as CRC, but also for diseases affecting other tissues, such as those that imply epithelial–neuronal communication^28^ and those affected by perturbations of intestinal permeability.^29^

The aims of this study were as follows: (1) to provide a reference transcriptomic data set for normal colon epithelium by profiling gene expression and AS, (2) to identify SNPs associated with variation in gene expression and AS (ie, QTLs), (3) to list traits for which identified QTLs contribute to SNP-based heritability, (4) to prioritize candidate effector genes, and (5) to provide a web-based resource to visualize the expression profiles and QTLs.

## Results

The University of Barcelona and University of Virginia genotyping and RNA Sequencing Project: A Novel Reference Data Set for Colon Tissue Transcriptome Analysis

The University of Barcelona and University of Virginia genotyping and RNA sequencing project (BarcUVa-Seq) cross-sectional study included 485 adult volunteers found to have an endoscopically healthy colon (ie, a normal colon without polyps or other lesions) from whom we collected superficial colon biopsy specimens and blood samples. Bulk RNA was isolated from biopsy samples and sequenced in several batches. Subjects were genotyped using the Illumina (San Diego, CA) OncoArray 500K beadchip,^30^ and genome-wide SNPs were imputed. After filtering the data to select for individuals with high-quality RNA-Seq and genotype samples (see the Materials and Methods section), we included data from 445 individuals, among whom 283 were female (64%). Biopsy specimens were obtained from sites along the ascending (n = 138; 31%), transverse (n = 143; 32%), and descending (n = 164; 37%) colon (Table 1). We profiled gene expression and alternative splicing and identified cis-acting eQTLs and sQTLs (see the Materials and Methods section).

**Table 1:** BarcUVa-Seq Data Set Descriptive

Total individuals, N	445
Sex, n (%)	
Female	283 (63.6)
Male	162 (36.4)
Age, *y*, means ± SD	60 ± 7.44
Colon anatomic location overall and stratified by sex, n (%)	
Ascending (right)	138 (31.0)
Female	86 (62.3)
Male	52 (37.7)
Transverse	143 (32.1)
Female	90 (62.9)
Male	53 (37.1)
Descending (left)	164 (36.9)
Female	107 (65.2)
Male	57 (34.8)

### Gene Expression and Alternative Splicing

**Figure 1:**
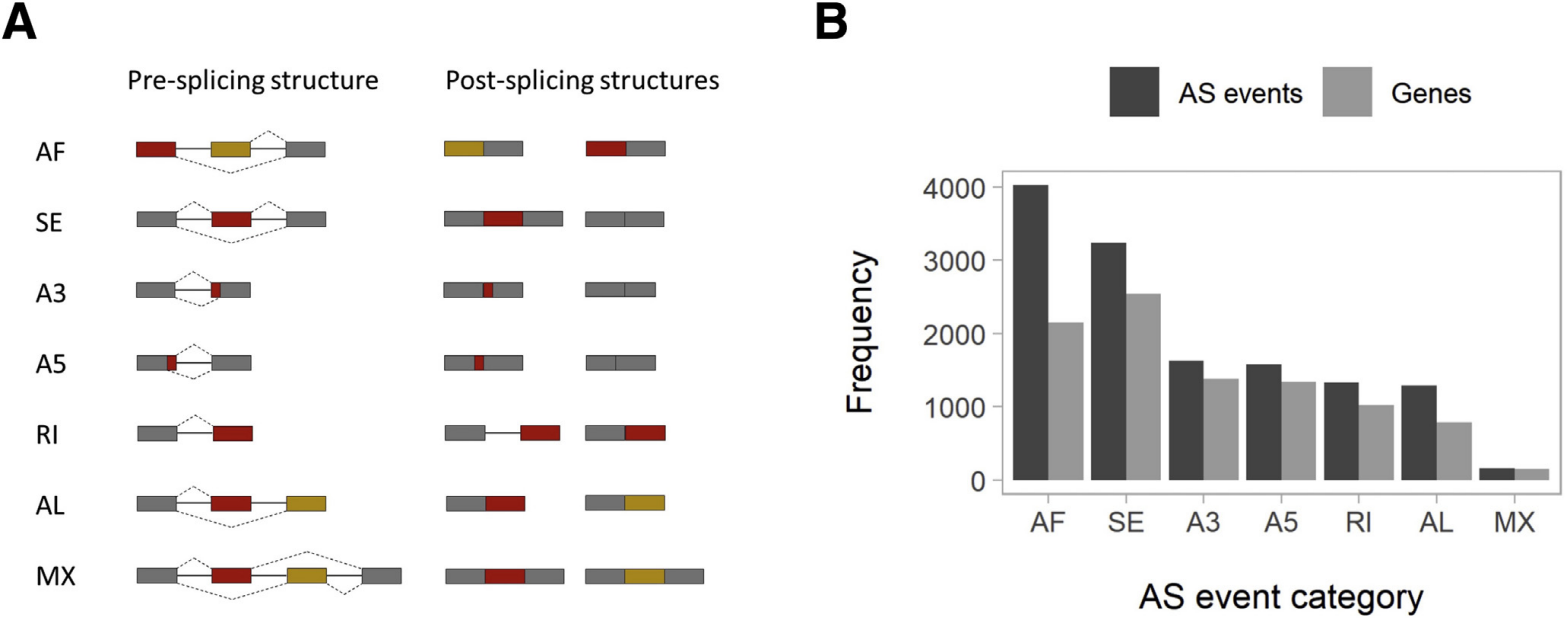
**Alternative splicing events.** (*A*) Scheme of gene and alternatively spliced transcripts structure in 7 AS categories: alternative first exons (AF), exon skipping (SE), alternative 3’ splice-site (A3), alternative 5’ splice-site (A5), intron retention (RI), alternative last exons (AL), and mutually exclusive exons (MX). Constitutive exons (ie, those maintained in all processed transcripts after splicing) are shown in gray. Exons in red or gold alternatively are present in processed transcripts after splicing. *Dashed line* indicates different splicing processing for a gene. (*B*) Frequency of AS events and genes by AS category. One gene can be processed according to different AS categories.

Expression was analyzed based on GENCODE (E;BL-EBI, Hinxton, UK) release 19 annotations.^31^ After filtering out features with low or no expression, 21,281 genes and 104,769 transcripts remained (see the Materials and Methods section). Gene and transcript abundances of interest can be visualized online (see the Colon Transcriptome Explorer [CoTrEx] section). We considered 13,243 AS events in 6178 genes after applying filters (see AS events annotations in Supplementary Table 1). We categorized AS events as follows: alternative first exons (30%), exon skipping (24%), alternative 3’ splice-site (12%), alternative 5’ splice-site (12%), intron retention (10%), alternative last exons (10%), and mutually exclusive exons (1%) (Figure 1, Table 2). Most genes had AS events from 1 or 2 categories, and few had AS events from up to 6 categories. In addition, as a complementary AS metric, we computed the abundances of 269,586 alternatively excised introns that were grouped in 73,313 clusters. Some introns (23%) were novel and 77% were annotated in 15,912 genes. We filtered introns by low expression or low complexity and considered only 42,808 intron clusters annotated in 8953 genes for sQTL analysis (see the Materials and Methods section).

**Table 2:** Description of AS Events and Genes by AS category

Event category	Total AS events, n (%)	Total genes, n (%)	AS events associated with sSNPs, n (%)
SE	3235 (24.43)	2542 (41.20)	316 (28.1)
AF	4023 (30.38)	2146 (34.78)	253 (22.5)
A3	1627 (12.29)	1378 (22.33)	140 (12.4)
A5	1579 (11.92)	1344 (21.78)	148 (13.2)
RI	1327 (10.02)	1022 (16.56)	126 (11.2)
AL	1292 (9.76)	785 (12.72)	259 (11.5)
MX	160 (1.21)	148 (2.40)	12 (1.1)
Overall	13,243 (100.00)	6170 (100.00)	1125 (100.0)

NOTE. A given gene can have AS events from up to 6 categories.

AF, alternative first exons; AL, alternative last exon; A3, alternative 3’ splice-site; A5, alternative 5’ splice-site; RI, intron retention; MX, mutually exclusive exons; SE, exon skipping.

### Transcriptomic Profiles Differ Between Colon Subsites

**Figure 2:**
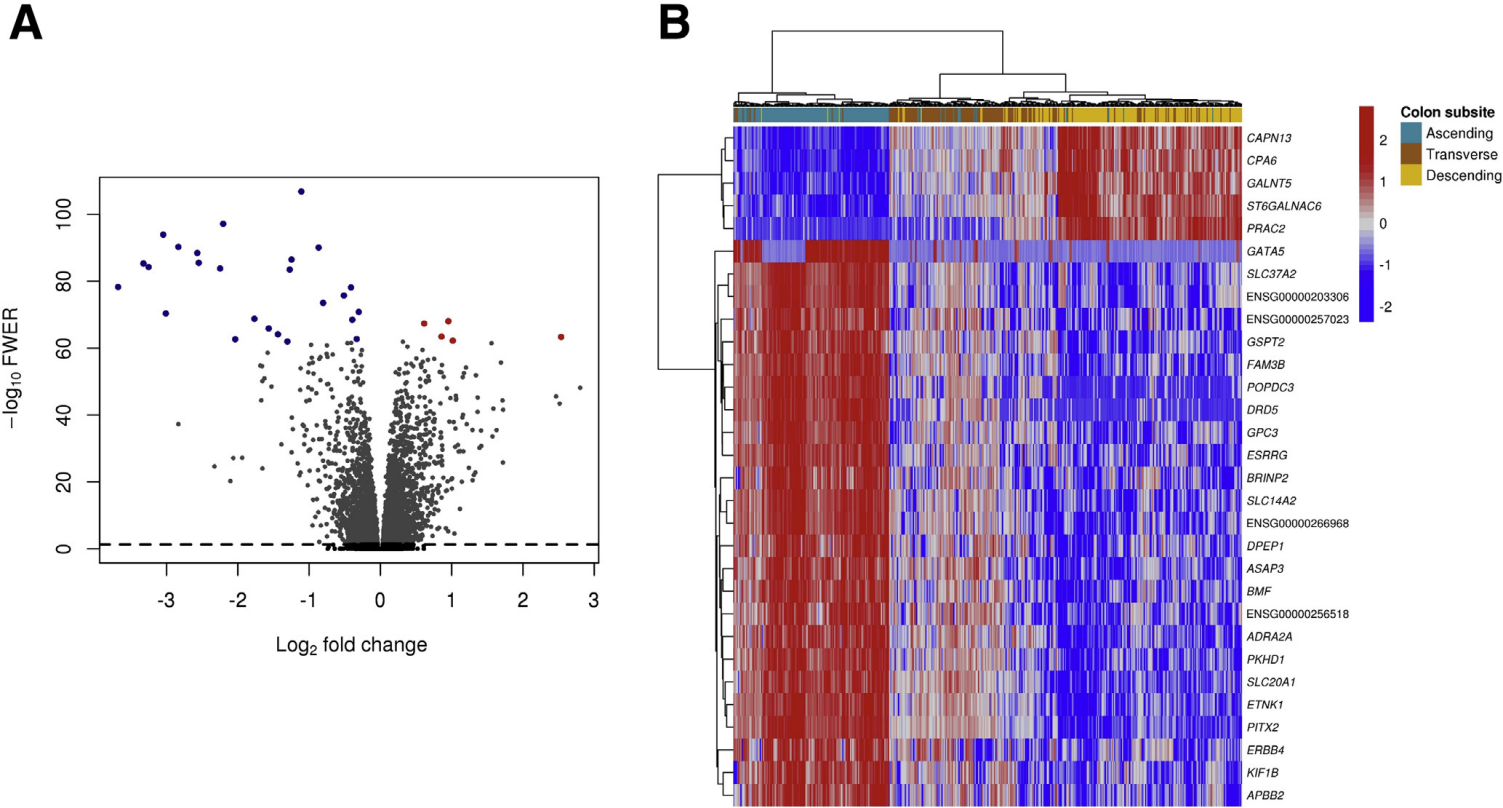
**Differential gene expression profiles across colon anatomic subsites.** (*A*) Volcano plot showing the distribution of gene log fold changes and statistical significance. Points above the *horizontal dashed line* represent genes considered significantly differentially expressed (FWER ≤ 0.05). Points in *red* and *blue* color represent genes over (red) and underexpressed (blue) following a consistent trend from ascending to descending colon (ie, overexpressed in transverse relative to ascending colon and overexpressed in descending relative to transverse). (*B*) Heatmap showing the expression profiles of the top 30 differentially expressed genes across colon subsites ranked by FWER-adjusted *P* values. Hierarchical clustering shows the similarity between genes (*rows*) and samples (*columns*) based on Euclidean distances.

We aimed to identify genes and splicing features that were expressed differentially across colon subsites, situating the transverse colon as an intermediate phenotype (see the Materials and Methods section). Overall, 4430 genes were expressed differentially between ascending, transverse, and descending subsites (family-wise error rate [FWER], ≤0.05), with absolute log fold changes of up to 3.7 (Figure 2*A*). Hierarchical clustering of the top 30 genes with the smallest FWER showed the transverse colon clustered with descending colon (Figure 2*B*). Full differential gene expression results are listed in Supplementary Table 2. Next, we tested whether genes expressed differentially across subsites were enriched for features in a wide array of curated gene sets, signatures, functional pathways, and ontologies. We found enrichment in a gene set associated with normal colon tissue transformation into adenoma, in pathways involved in drug metabolism, and in other biological processes such as antimicrobial humoral response. Full enrichment results are listed in Supplementary Table 3. For splicing, we found 236 genes with different relative abundances of AS events (false-discovery rate [FDR], ≤0.05) (Supplementary Tables 4 and 5) and 280 genes with different relative abundances of excised introns between the ascending and descending colon (FDR, ≤0.05) (Supplementary Table 6).

### Identification of eQTLs and sQTLs

**Figure 3:**
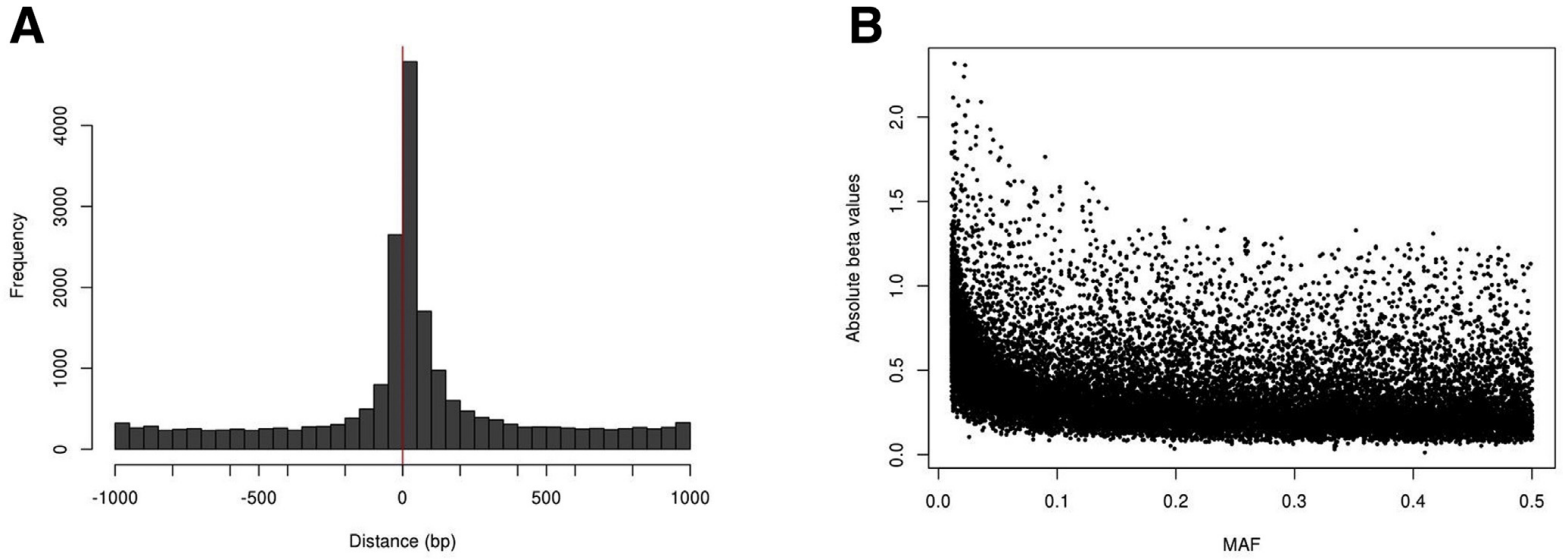
**eQTLs features.** (*A*) Distribution of distances between eSNPs location and corresponding eGenes TSS. (*B*) Distribution of absolute beta values (slope associated with the nominal *P* value of association) of eQTLs and eSNPs minor allele frequencies (MAF). These variables were not correlated (r = 0.14).

**Figure 4:**
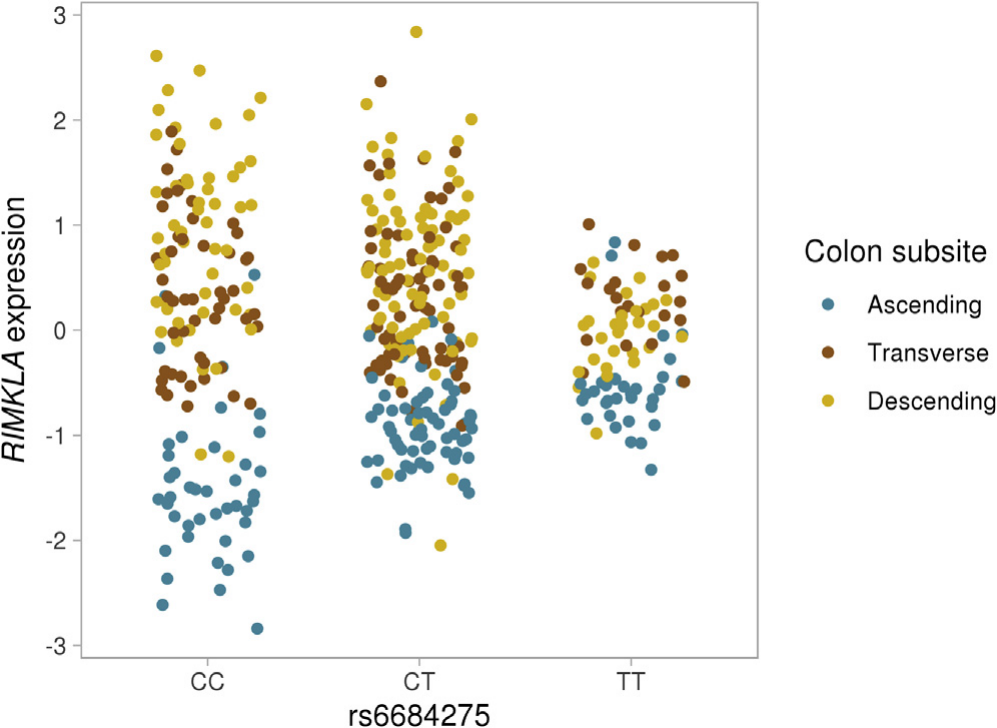
**Example of eQTLs interacting with colon subsite.** Distribution of expression level (inverse normal transformed trimmed means of M values) of *RIMKLA* by rs6684275 genotype and colon subsite.

We identified 11,739 eQTLs (Q value ≤ 0.05) including 11,427 unique SNPs (eSNPs) associated with the expression of 11,739 genes (eGenes) (Supplementary Table 7). Most eSNPs were associated with a single eGene, but we found eSNPs associated with up to 6 eGenes. Neither the location of the eSNPs relative to the gene transcription start site (TSS) nor the allele frequency were associated with the eSNP effect (Figure 3). eQTLs can be explored on the CoTrEx web application (see the Colon Transcriptome Explorer section). Full eQTL summary statistics are publicly available (see the Data availability statement). In addition, we performed eQTL interaction analysis for colon subsites (ascending vs descending) and found 26 eQTLs with a Q value of 0.05 or less (Supplementary Table 8). The eQTL rs6684275-*RIMKLA* showed an inverse association in the ascending colon compared with the descending colon (Figure 4).

**Figure 5:**
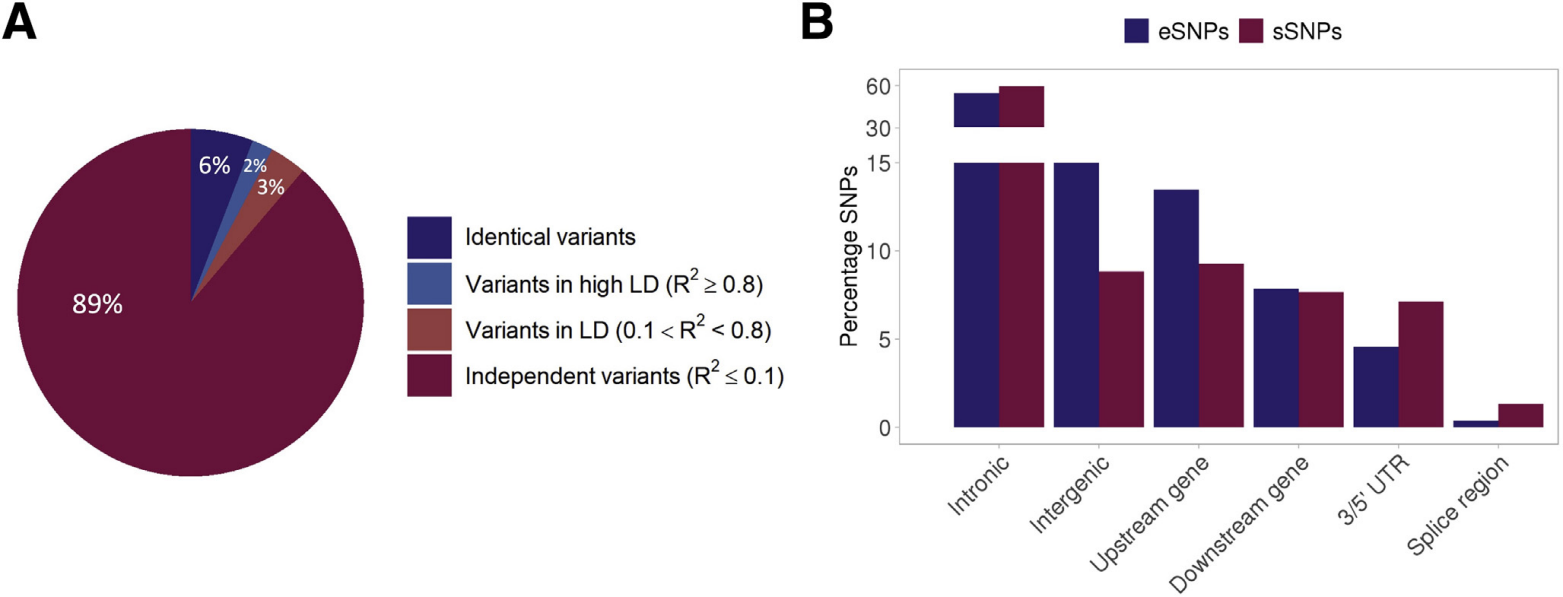
**Colocalization among sSNPs and eSNPs and genomic region annotation.** (*A*) Percentages of colocalization patterns among sSNPs and eSNPs in common genes according to measures of LD R^2^. (*B*) Percentages of eSNPs and sSNPs at specific genomic regions, note that the plot is gapped between 15% and 30% and rescaled between 30% and 60% to show the differences in the categories with the lowest representation. UTR, untranslated region.

Next, we mapped 1125 sQTLs (Q value ≤ 0.05) including 1122 unique SNPs (sSNPs) associated with 1125 genes (sGenes) (Supplementary Table 9). The proportions of AS categories among SNP-associated AS events were similar to those found for total AS events (Table 2). Although we found 82% of sGenes among eGenes, only 8% of sGenes shared the same genetic variants with eGenes (6%) or harbored variants in high linkage disequilibrium (LD R^2^ > 0.8) with eSNPs (2%) (Figure 5*A*). In addition, we identified an additional set of 1062 sQTLs (Q value ≤ 0.05) of 1058 sSNPs associated with clusters of excised introns in 1062 genes (Supplementary Table 10) and observed that 40% of these sGenes were in common with sGenes associated with AS events. sQTLs can be explored on the CoTrEx web application (see Colon Transcriptome Explorer section), and full summary statistics are publicly available (see Data availability statement).

### Replication and Meta-Analysis With GTEx

**Figure 6:**
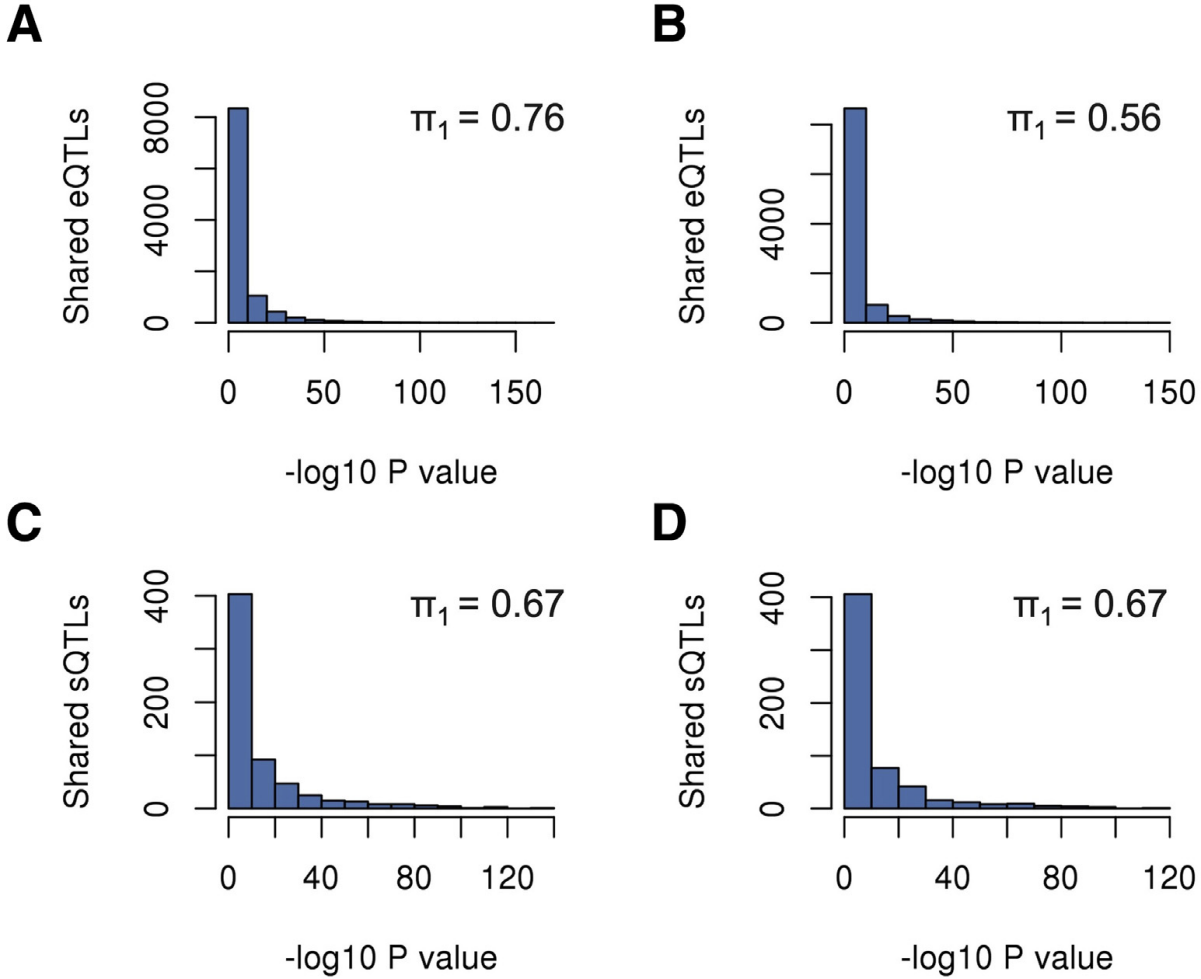
**Replication analysis of eQTLs/sQTLs with GTEx v8 colon data.** The value of the π1 statistic is shown. The distribution of *P* values is shown for (*A*) transverse colon eQTLs, (*B*) sigmoid colon eQTLs, (*C*) transverse colon sQTLs, and (*D*) sigmoid colon sQTLs.

We performed replication and meta-analyses using data from the GTEx project v8.^8^ For replication analysis, we used samples from the sigmoid and transverse colon (n = 318 and n = 368, respectively). For the replication of eQTLs, we downloaded the list of GTEx eQTLs (see the Materials and Methods section). For the replication of sQTLs we used GTEx transcript expression data for computing AS events as well as SNPs for computing sQTLs using the same approach considered for BarcUVa-Seq data (Supplementary Tables 11 and 12). We explored the *P* value distributions between BarcUVa-Seq and GTEx colon data sets and computed the π_1_ statistic^32^ (Figure 6). For eQTLs, a higher replication value (π_1_ = 0.76) was obtained for GTEx transverse colon than for sigmoid colon (π_1_ = 0.56). For sQTLs the same replication statistic was obtained for both GTEx colon tissue data sets (π_1_ = 0.67).

**Figure 7:**
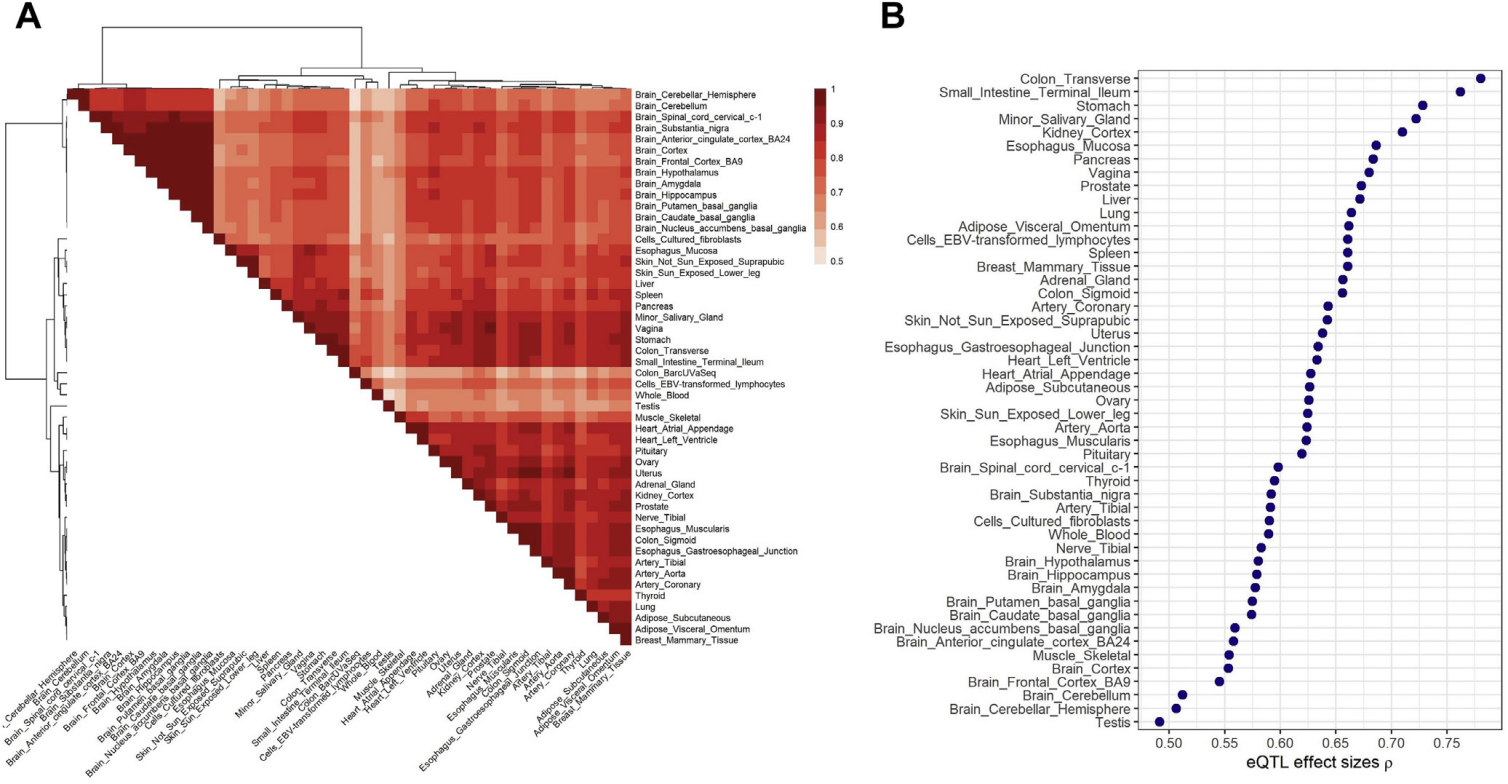
**Meta-analysis with GTEx v8 tissues.** (*A*) Clustering of BarcUVa-Seq and GTEx v8 tissues based on pairwise Spearman correlation of eQTL effect sizes derived from mashr meta-analysis. We only considered significant (FDR ≤ 0.05) and active (local false sign rate [LFSR] ≤ 0.05) eQTLs. (*B*) Spearman correlation of eQTL effect sizes between BarcUVa-Seq and GTEx v8 tissues. eQTL effect sizes were derived from mashr meta-analysis. We only considered significant (FDR ≤ 0.05) and active (LFSR ≤ 0.05) eQTLs.

We performed a meta-analysis of BarcUVa-Seq eQTLs with the full GTEx v8 data set (n = 49 tissues) using a multivariate adaptive shrinkage approach.^33^ Hierarchical clustering of pairwise correlations on the resulting effect sizes showed that BarcUVa-Seq eQTLs from colonic mucosa clustered with GTEx eQTLs from transverse colon and terminal ileum (Figure 7*A*). The correlations between BarcUVa-Seq eQTL effect sizes and all GTEx tissues showed that transverse colon, terminal ileum, stomach, minor salivary gland, and kidney cortex are the GTEx tissues with highest correlation (ρ > 0.7) (Figure 7*B*).

### Annotation and Functional Enrichment Analyses

We observed eSNPs and sSNPs distributed in patterns similar to each other across the following genomic regions: introns, intergenic regions, upstream and downstream gene regions, 3’ and 5’ untranslated regions and splice regions (including donor and acceptor variants). Intronic variants were the most common from both types of SNPs. Intergenic and upstream regions harbored higher proportions of eSNPs than sSNPs, and splice and untranslated regions harbored higher proportions of sSNPs than eSNPs (Figure 5*B*). Functional consequences also were assessed: most SNPs were not classified, but a small proportion of SNPs were classified as nonsense, start loss, frameshift, canonical splice site, missense, or synonymous variants (Supplementary Table 13).

**Figure 8:**
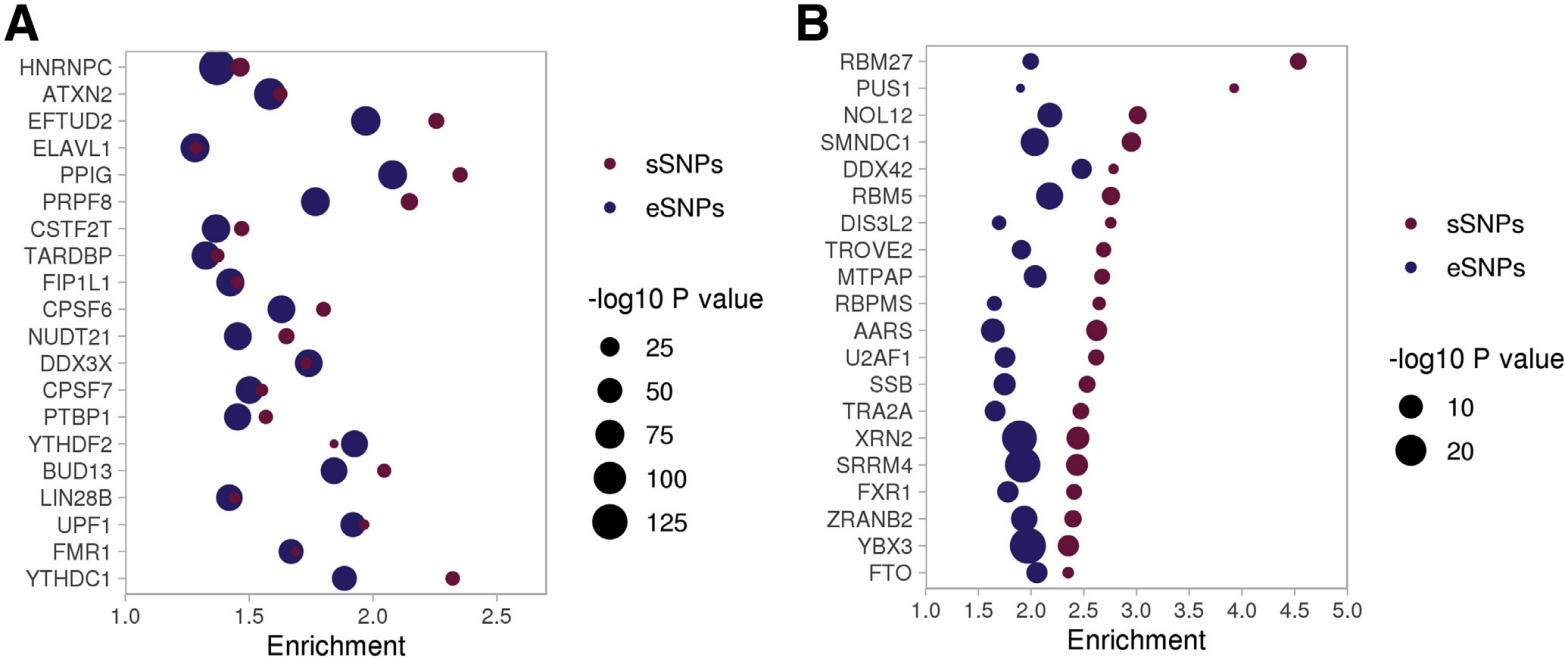
**Enrichment of eSNPs/sSNPs in binding sites across the genome of RBPs.** (*A*) The top 20 RBP with the lowest enrichment *P* values for eSNPs. (*B*) The top 20 RBPs with the highest enrichment values for sSNPs (*P* value < .05).

Next, we performed enrichment analysis at regulatory regions (open chromatin regions, active enhancers, superenhancers, and transcription factor binding sites) using data derived from colon cell lines as well as from normal and cancerous colon tissue. We found significant enrichment (*P* value ≤ .05) in all types of regulatory regions for both eSNPs and sSNPs. In addition, we looked for enrichment in target sites distributed across the genome of 170 RNA-binding proteins (RBPs). The top 20 RBPs with the lowest *P* values for eSNP enrichment are included in Figure 8*A*. Of those RBPs, 15 also were among the top 20 RBPs most enriched for sSNPs. In both cases, the heterogeneous nuclear ribonucleoprotein C was the RBP with the most significant enrichment. The RBPs with highest enrichment values for sSNPs are included in Figure 8*B*. We observed sSNPs enriched at binding sites of spliceosome constituents such as the splicing factor U2 small nuclear RNA auxiliary factor 1. Full enrichment results are listed in Supplementary Table 14.

### Phenotype Heritability Enrichment and Colocalization Analyses

**Figure 9:**
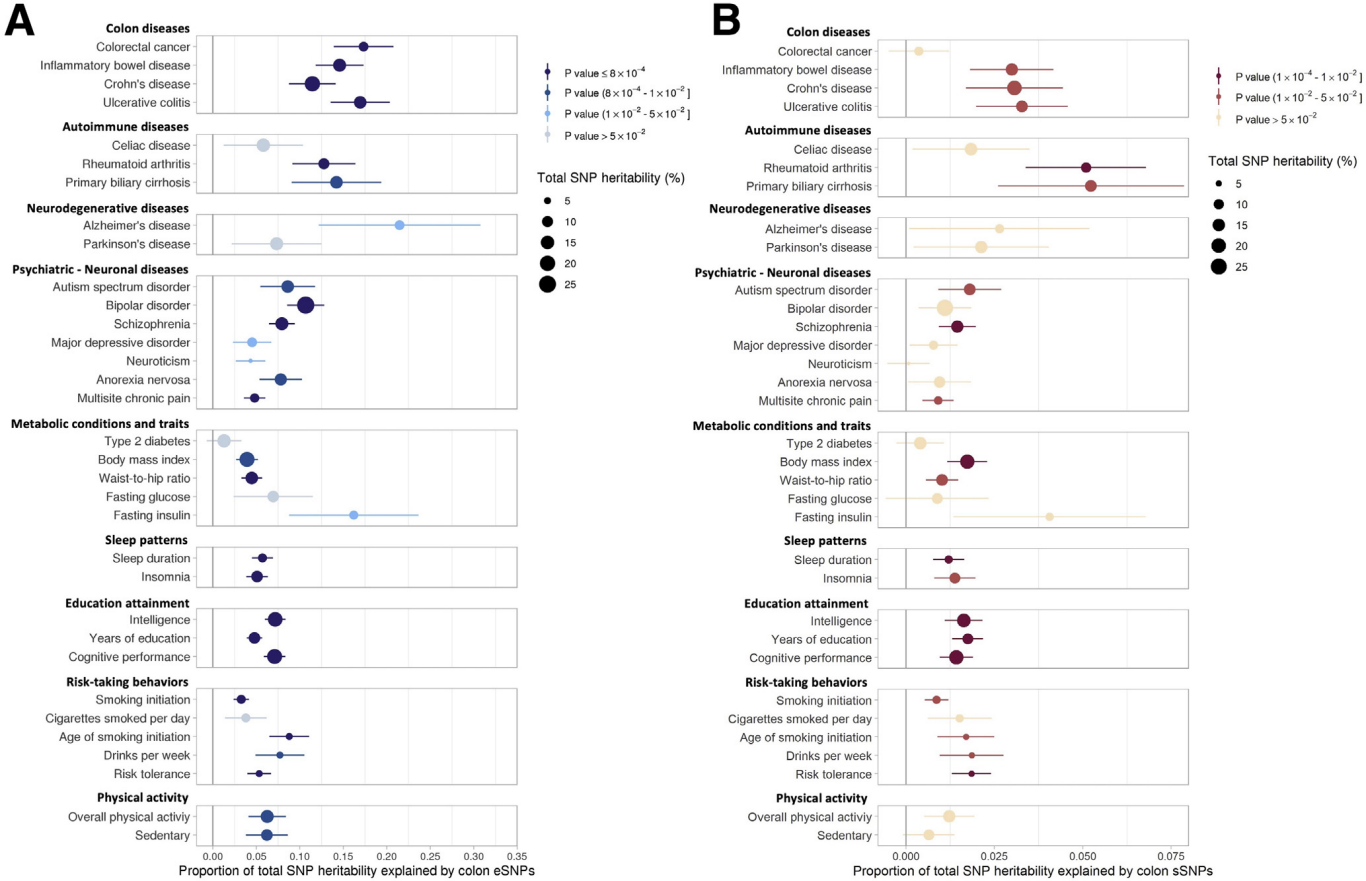
**BarcUVa-Seq QTL enrichment results for total SNP heritability of 33 complex traits and diseases related to colon tissue.** (*A*) Proportion of total SNP heritability explained by eSNPs is shown on the x axis, along with error bars. The size of the points indicates the percentage of the total SNP heritability out of the total heritability of the phenotype. (*B*) Proportion of total SNP heritability explained by sSNPs is shown on the x axis, along with error bars. The size of the points indicates the percentage of the total SNP heritability out of the total heritability of the phenotype.

To quantify the ability of BarcUVa-Seq QTLs to explain a phenotype’s genetic risk loci, we analyzed eSNPs/sSNPs in the context of their potential contribution to total SNP-based heritability estimates of multiple complex traits. SNP-based heritability is the heritability of traits captured by SNPs in a SNP array in the context of a genome-wide association study (GWAS). We performed SNP-based heritability enrichment tests in 63 complex diseases and traits that we considered a priori to influence or be influenced by colon homeostasis. We observed that eSNPs were enriched in the SNP-based heritability estimation of 20 diseases or traits after Bonferroni adjustment (*P* value ≤ 8 × 10^-4^) and 31 diseases or traits at an unadjusted *P* value ≤ .01. SNP-heritability enrichments for 33 traits and diseases are included in Figure 9*A*, and full results are listed in Supplementary Table 15. BarcUVa-Seq eSNPs explained 17% of the total SNP-based heritability of CRC (*P* value = 9 × 10^-8^), which accounts for 10% of the phenotype (based on a recent GWAS study^34^). Interestingly, eSNPs also were enriched in the SNP-based heritability estimation of psychiatric–neuronal disease, such as schizophrenia, bipolar disorder, and multisite chronic pain. BarcUVa-Seq sSNPs were enriched in the SNP-based heritability estimation of 10 diseases and traits at a *P* value ≤ .01, but no enrichments were statistically significant after Bonferroni adjustment (Figure 9*B* shows 33 representative traits or diseases, Supplementary Table 15 has the full list of results). BarcUVa-Seq sSNPs explained 3% of the total SNP heritability of ulcerative colitis (*P* value = .02), which accounts for 13% of the phenotype (Figure 9*B*).

**Figure 10:**
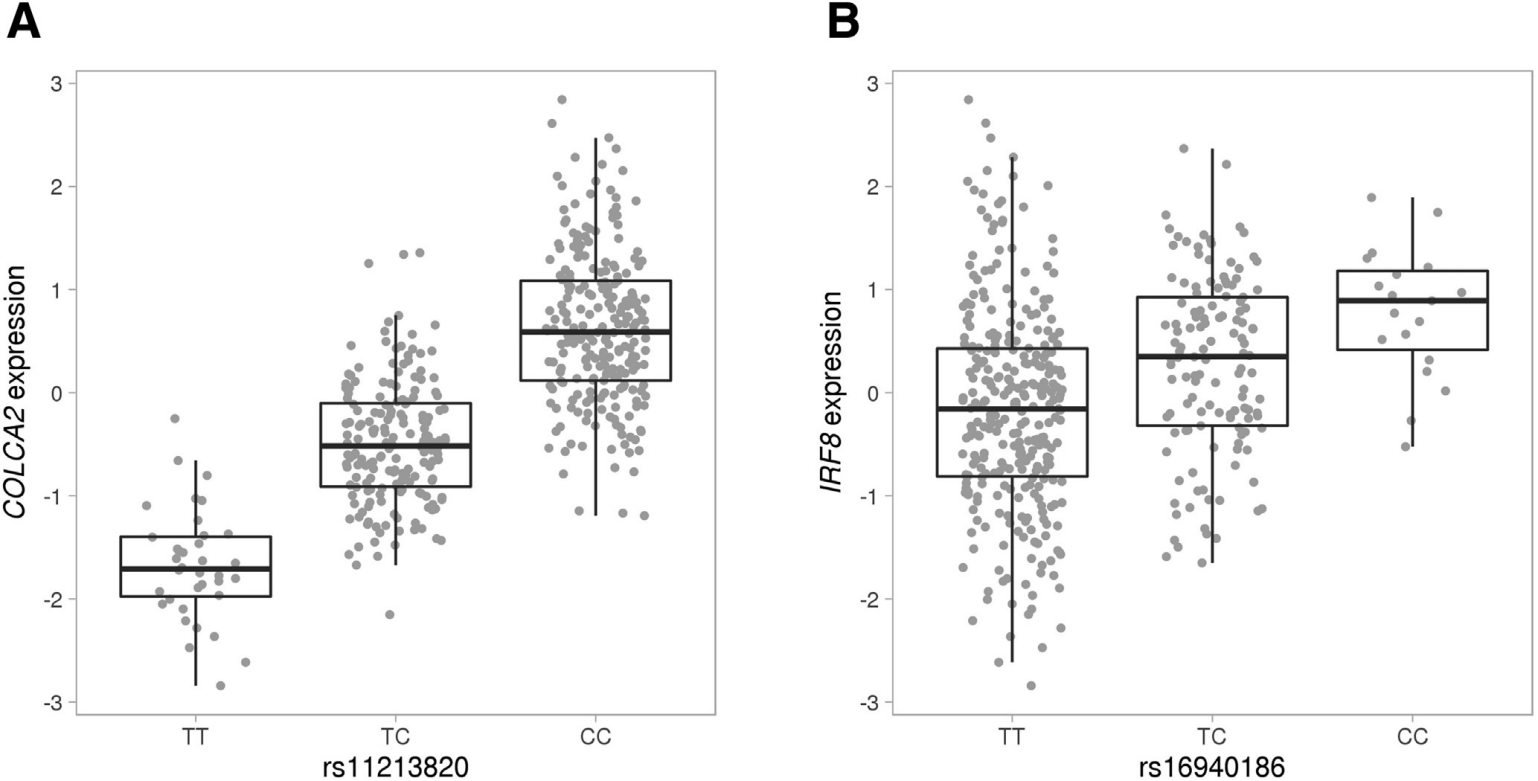
**The top eQTLs of the genes with the highest regional colocalization probability for CRC and inflammatory bowel disease.** (*A*) Expression level (inverse normal transformed trimmed means of M values [TMMs]) of *COLCA2* by genotype of the eSNP rs11213820. (*B*) Expression level (inverse normal transformed TMMs) of *IRF8* by genotype of the eSNP rs16940186.

Subsequently, to nominate candidate genes at GWAS-identified genetic risk loci, we performed colocalization analyses for the complex traits and diseases that passed Bonferroni correction for SNP-based heritability analysis for BarcUVa-Seq eSNPs. The regional colocalization probability is used as a proxy for the gene’s causality, that is, to quantify the probability that an eQTL and a GWAS signal share the same causal variant.^35^ In the case of CRC, we identified 13 genes with regional colocalization probability greater than 0.9, including known risk genes such as *COLCA1* and *COLCA2*,^6^ as well as other less-well-described genes such as *ANKRD36*. In the case of inflammatory bowel disease, we identified 6 genes with a regional colocalization probability greater than 0.9, such as *IRF8* and *RGS14* (Figure 10). Full results are available in the Supplementary Data.

### Colon Transcriptome Explorer

**Figure 11:**
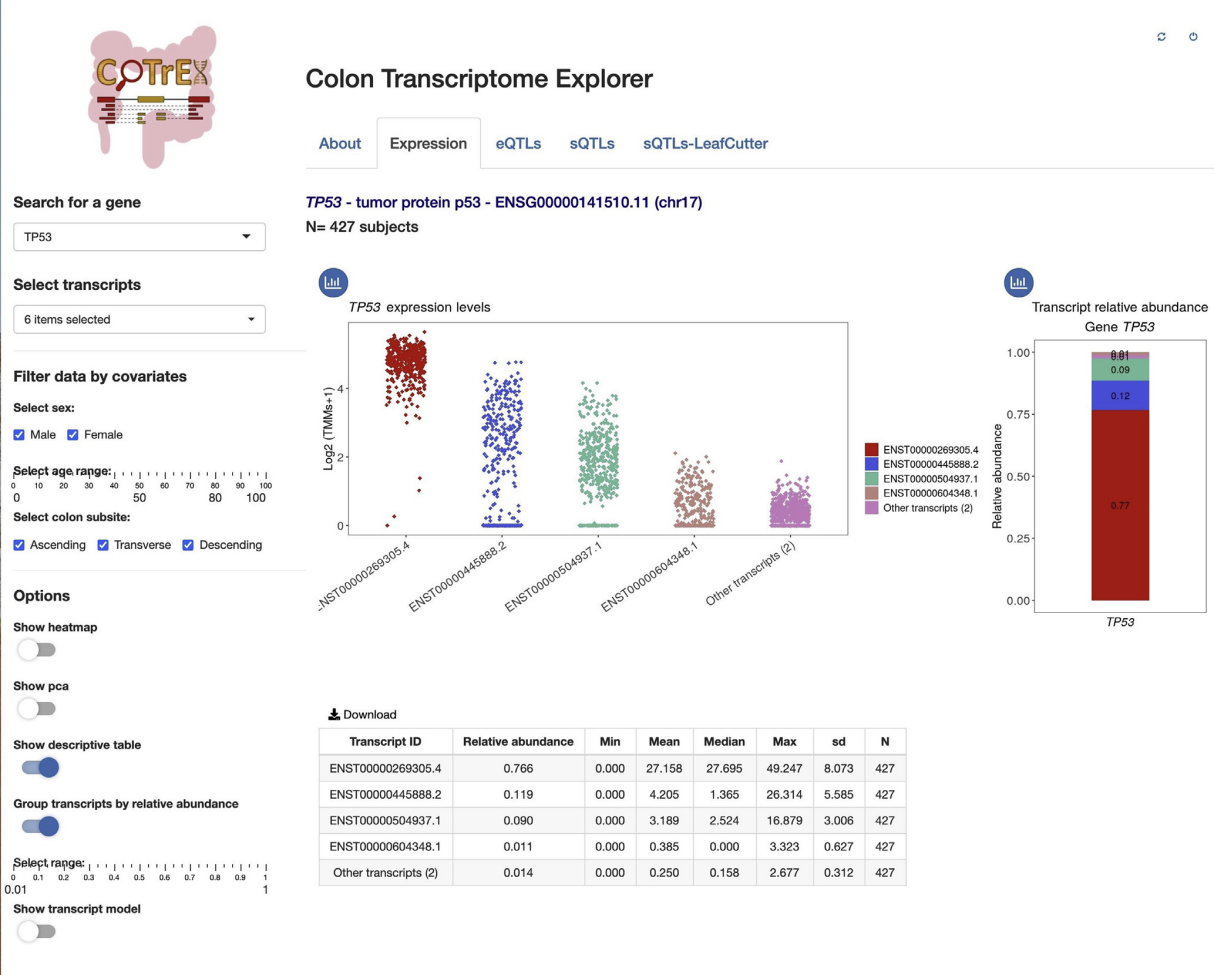
**Overview of the expression tab of CoTrEx.** As an example, the transcript expression values and relative abundances of the *TP53* gene are shown, along with different display options.

Gene and transcript abundances for the BarcUVa-Seq data set, as well as eQTLs/sQTLs, have been loaded into the web-based visualization resource CoTrEx. This tool facilitates searches for genes and transcripts of interest for their visualization in customizable plots, such as a strip chart, heatmap, and principal component analysis (PCA) plots. The interactive application includes different options for filtering and coloring the data by covariates. Figure 11 shows an example in the Expression tab. CoTrEx is freely available online at http://barcuvaseq.org/cotrex.

## Discussion

In the present study we analyzed a large data set (BarcUVa-Seq) comprising germline SNPs and transcriptome profiles from mucosal biopsy specimens of ascending, transverse, and descending colon collected from 445 healthy living individuals. Differential expression patterns were identified across colon subsites. We profiled 11,739 eQTLs comprising 11,427 unique SNPs associated with the expression of 11,739 genes. In addition, we identified 13,243 AS events from 7 distinct AS categories and identified 1125 AS events in 1125 genes associated with 1122 unique SNPs (sQTLs). These eQTLs/sQTLs frequently were intronic and enriched in regulatory regions. We showed how these are useful for annotation of GWAS-identified risk loci and prioritization of candidate effector genes. Moreover, we replicated and meta-analyzed our QTLs with GTEx v8 data. Finally, we built an interactive web resource to explore the expression profiles and QTLs of the BarcUVa-Seq data set.

In contrast to BarcUVa-Seq, the GTEx project provided RNA-Seq data on sigmoid and transverse colon tissue from post-mortem subjects and extracted RNA from full-thickness and muscularis-only sections.^8^^,^^36^ Our novel BarcUVa-Seq data set overcomes some of the limitations of the GTEx colon data sets. BarcUVa-Seq samples were collected as superficial mucosal biopsy specimens in living subjects undergoing colonoscopy, which provide an optimal representation of the normal physiology of the colon epithelium. Moreover, they included subsites of the large intestine not assessed previously. Together with the enrichment of colon epithelial cells in superficial biopsy specimens, inclusion of ascending, transverse, and descending colon samples make BarcUVa-Seq a unique colon transcriptome data set.

Next-generation RNA-Seq data provide estimates of AS. Although long-read sequencing technologies can provide transcriptomic profiles with full-length isoform information, such technologies have lower base-level fidelity and are less feasible in large population-based studies at their current cost.^11^ In this study we used 2 complementary methods to provide a comprehensive profile of AS. The frequencies of genes with specific AS patterns that we identified in colon tissue are similar to those described in other tissues, where genes with exon skipping events were the most frequent.^17^ Predicting AS events helps generate hypotheses about specific molecular mechanisms involved in post-transcriptional modifications. In contrast to profiling individual transcripts to characterize the transcriptome, AS events group transcripts with similar structure. However, the profiles of annotated AS events are sensitive to the choice of transcript annotations,^11^ and other measures of AS, such as clusters of excised introns, complement the characterization of AS events.^13^

Regarding colon location, transcriptomic differences between subsites in normal colon have been described previously,^37^ including gene expression differences in genes from the cytochrome P450 family. In addition, different AS events have been identified between CRC tumors located in the ascending and descending colon.^38^ Indeed, tumor distribution across the colon has been associated with differential mutation and immune profiles, prognosis, and treatment response.^39^^,^^40^ In this study, we identified a subset of genes expressed differentially between colon subsites that are involved in molecular pathways related to lipid, xenobiotic, and drug metabolism, and a subset of genes involved in antimicrobial response. We observed that the gene expression profile of transverse colon tissue was more similar to the descending than to the ascending colon, which was unexpected based on embryologic origin and adult blood supply. Differential gene expression across the colon may reflect differences in cell type composition because we find gene markers of different cell types of the colon epithelium shown by single-cell RNA-Seq studies.^41^, ^42^, ^43^ For instance, using our data, we confirmed that goblet cell markers defined elsewhere,^41^ such as *MUC2* and *TFF3*, are overexpressed in descending colon (Supplementary Table 2), which supports previous findings that have shown that goblet cell content increases caudally from duodenum to distal colon.^44^ Differential expression also may be influenced by differential exposure owing to variability in luminal content along the length of the colon, including microbial communities.^43^

We identified eQTLs and sQTLs assumed to participate in the transcriptional regulation of colon epithelium via cis mechanisms. These had strong replication in the transverse colon from GTEx v8 and were more similar to tissues with a high proportion of mucosa (eg, terminal ileum, stomach, and salivary gland) than others from GTEx v8, showing the robustness of BarcUVa-Seq data. The lower replication value in sigmoid colon may be owing to the higher proportion of muscularis in this tissue.^8^^,^^36^ We found fewer sGenes than eGenes, partly because the number of genes that showed splicing variability was lower than genes with expression variability. In addition, we had lower power to detect expression for transcripts than for genes at our depth of coverage. We found similar distributions of eSNPs/sSNPs around gene TSSs, as well as across estimated effect sizes, genomic locations, and functional consequences. We observed a high proportion of sGenes among eGenes, as reported elsewhere.^24^^,^^25^ Although they can colocalize, eQTLs and sQTLs usually are independent.^27^ sQTLs add information to eQTLs as they associate SNPs with changes in relative use of specific sets of transcripts sharing a common structure and post-transcriptional mechanism.

In this study, we showed that regulation of gene expression and AS is associated with tissue-specific epigenetic variations, including chromatin remodeling and histone modifications.^45^ The dysregulation of these features has been associated with initiation and progression of diseases such as CRC.^45^^,^^46^ We showed that normal colon eSNPs/sSNPs are present at many important regulatory regions marked by epigenetic signatures, such as open chromatin and proximal enhancers of both normal and malignant colon tissue. In addition, we identified specific RBPs and transcription factors as potential regulators of AS in normal colon.

We provide a comprehensive profile of AS for normal tissue along colon subsites in living subjects. We described differential gene expression and splicing between the ascending and descending normal colon, which involved genes of immune response and drug metabolism. We expanded the number of colon QTLs and assessed eQTL interaction with colon subsites. In addition, we observed that colon eQTLs/sQTLs contributed to the SNP-based heritability of brain-related traits and disease, supporting a model of epithelial–neuronal communication along the gut–brain axis.^28^ Thus, our QTL catalog may be of potential interest for researchers investigating traits and diseases that do not primarily affect the colon, but other organs. It is important to note that these results could reflect a common regulation of expression between tissues. In addition, colocalization alludes to potential molecular mechanisms associated with risk loci, but may not prove to be directly causal.

Overall, our findings provide evidence of the regulation of gene expression and alternative splicing in the colon as potential underlying mechanisms of genetic risk loci and should serve as a rich resource for the research community.

## Methods

### Sample Collection

Subjects included in the study (n = 445; 64% females) had a mean age of 60 years, were almost all of European ancestry, and received an indication for colonoscopy after a positive fecal immunochemical test result (hemoglobin level, >20 mg Hb/g) or by direct referral by their medical doctor. Subjects had no lesions at colonoscopy and no history of polyps or CRC. Non-neoplastic colon mucosa biopsy specimens were obtained endoscopically from the ascending (n = 138; 31%), transverse (n = 143; 32%), and descending (n = 164; 37%) colon (Table 1). Peripheral blood samples also were collected. Informed consent was obtained from all participants. The corresponding study protocol was approved by the Bellvitge University Hospital Ethics Committee (PR073/11 and PR286/15) and followed national and international directives on ethics and data protection. More information about the BarcUVa-Seq project can be accessed online at https://barcuvaseq.org. All authors had access to the study data and reviewed and approved the final manuscript.

### RNA-Seq Library Preparation and Sequencing

RNA was extracted from frozen tissue using the mirVana kit (Thermo Fisher Scientific, Waltham, MA) after homogenization using the Minilys bead mill (Bertin Instruments, Montigny le Bretonneux, France). The RNA was DNAse treated and concentrated using the RNA Clean and Concentrator-5 kit (Zymo Research, Irvine, CA). Quantification of total RNA was executed using a Qubit Fluorometer (Invitrogen, Walthan, MA). An Agilent (Santa Clara, CA) 2100 Bioanalyzer or TapeStation was used to assess quality. For library preparation, the Illumina TruSeq Stranded Total RNA Library Prep Gold kit was used. Libraries were tagged with unique adapter indexes. Final libraries were validated on the Agilent 2100 Bioanalyzer, quantified via quantitative polymerase chain reaction, pooled at equimolar ratios, diluted, denatured, and loaded onto an Illumina HiSeq 2500 (high-output mode), for batches 1–7, or a NovaSeq 6000, for batch 8, instruments using a paired-end flowcell.

### RNA-Seq Data Processing

Low-quality bases, sequencing adapters, and ribosomal RNA of raw sequences were trimmed from RNA-Seq reads using BBTools suite (Joint Genome Institute, Berkeley, CA).^47^ FastQC (Babraham Bioinformatics, Cambridge, UK)^48^ was used for quality control. Trimmed reads were aligned against human transcriptome using the Genome Reference Consortium human reference 37 assembly (GRCh37/hg19) with the Spliced Transcripts Alignment to a Reference (STAR, Cold Spring Harbor Laboratory, Cold Spring Harbor, NY) software in 2-pass mode^49^ using GENCODE (EMBL-EBI, Hnxton, UK) release 19 annotations, which include a total of 57,952 genes and 196,667 transcripts.^31^ We only included samples with a depth of coverage greater than 10 million mappable paired-end reads, a multimapping rate lower than 15%, and a unique mapping rate greater than 80%. The mean library size was 32M (SD, 8.5M). Gene and transcript expression were quantified with RSEM (University of Wisconsin-Madison, Madison, WI).^50^ Genes and transcripts with fewer than 6 and 3 counts, respectively, in less than 10% of the samples were considered not expressed and filtered out. Trimmed mean of M values were computed from counts to correct for library size and RNA composition.

### Genotype Data Processing

Genotyping of approximately 400,000 SNPs was performed with the Illumina OncoArray BeadChip.^30^ We only included samples with a genotyping rate greater than 95%. The following aspects also were assessed before imputation: duplication and relatedness greater than 0.8, missing rate per SNP greater than 0.1, missing rate per sample greater than 0.1, sex concordance (genetic and reported sex), heterozygosity: means ± 4 SD and Hardy–Weinberg disequilibrium *P* value less than 1 x 10^-^^4^. We obtained allelic dosages from 39,117,105 and 1,228,035 SNPs for autosomes and chromosome X, respectively, using SHAPEIT (University of Oxford, Oxford, UK)^51^ for phasing and Minimac 3 (University of Michigan, Ann Arbor, MI)^53^ for imputation with The Haplotype Reference Consortium panel on the Michigan Imputation Server.^52^ SNPs with an imputation quality of R^2^ less than 0.7 or minor allele frequency (MAF) less than 1% were excluded, resulting in 6,804,675 and 183,788 SNPs for autosomes and chromosome X, respectively. Allelic dosages were used for subsequent QTL analyses. SNP IDs were annotated using dbSNP version 142.^53^ Principal components of genetic data were obtained with PLINK 1.9 (Complete Genomics, Mountain View, CA).^54^ We checked that both genotype and RNA-Seq samples had been labeled correctly and belonged to the same individual using Picard Tools CheckFingerprint (Broad Institute, Cambridge, MA).

### Alternative Splicing Profiling

For quantifying AS, we used 2 complementary methods that provide the relative abundance (ie, percent splicing index [PSI]) of specific AS features. Seven types of AS events were determined based on GENCODE version 19 annotations with SUPPA2 (Catalan Institution for Research and Advanced Studies, Barcelona, Spain).^12^ In this case, the PSI reflects the proportion of transcripts of a given gene showing a specific AS event (ie, inclusion transcripts) of the total transcripts of the gene.^11^ This metric was calculated with SUPPA2 for each AS event by dividing the expression levels of the inclusion transcripts by the total expression levels of all transcripts of the gene. We kept AS events in which the median PSI for all samples was between 0.05 and 0.95 (see AS events annotations in Supplementary Table 1). As a complementary approach, we used LeafCutter (Stanford University, Stanford, CA)^13^ following the analysis procedure described elsewhere^8^ to compute the relative abundance of alternatively excised introns.

### Differential Gene Expression and Splicing Analysis

Differential gene expression analysis was performed using a quasi-likelihood F-test implemented in the R package edgeR (Garvan Institute of Medical Research, Parkville, Australia).^55^ Ward’s minimum variance method with Euclidean distances was used for hierarchical clustering. For differential splicing analysis, normalized PSI values of AS events were fitted in a linear model adjusted for sex, age, and sequencing batch using the R package limma (University of Melbourne, Parkville, Australia).^56^ The function *diffSplice* was used to perform an F test to find the differences between AS event log-fold-changes of a gene and yield a single gene-level *P* value. T tests for individual AS events also were performed with *diffSplice*. Differential use of excised introns was performed with LeafCutter,^13^ adjusting for sex, age, and sequencing batch. Functional enrichment analysis was performed with FUMA *gen2func* (University Amsterdam, Amsterdam, The Netherlands)^57^ using differentially expressed genes with FWER of 0.05 or less. FWER values were estimated for correcting for multiple testing using a Bonferroni correction.

### eQTL/sQTL Mapping

We mapped QTLs within 1 Mb of the TSSs for given genes and assumed QTLs influenced expression of nearby genes via cis mechanisms. For QTL identification we used FastQTL (University of Geneva Medical School, Geneva, Switzerland) version 2.0.^58^ We applied an inverse normal transformation on gene trimmed means of M values and PSI values, which mitigates the effect of outliers and normalizes the expression distribution across samples. We adjusted the models for age, sex, sequencing batch, tissue anatomic location, genetic ancestry (2 principal components), and probabilistic estimation of expression residuals factors,^59^ which capture the effects of unknown confounding variables. We chose the number of probabilistic estimation of expression residuals factors that maximized the discovery of eGenes/sGenes. FDR (Storey and Tibshirani procedure) was computed with R package qvalue (Princeton University, Princeton, NJ).^60^ For colon subsite eQTL interaction analysis we used the FastQTL version 2.0 interaction mode.^57^

### Replication and Meta-Analysis With GTEx Data

For replication analysis, we estimated π_1_^33^ with the R package qvalue.^60^ This statistic reflects the proportion of true positives among BarcUVa-Seq QTLs that also were detected by the corresponding QTL analysis in GTEx v8. Following a common approach described elsewhere,^8^ we only included associations involving the SNP with the lowest *P* value for each gene to avoid including many SNPs in LD. For meta-analysis, full GTEx v8 eQTL summary statistics (n = 49 tissues) were downloaded from the Google Cloud Platform (Mountain View, CA) under gtex-resources. We used a multivariate adaptive shrinkage approach using the R package mashr (University of Chicago, Chicago, IL)^33^ following the same analytic pipeline described elsewhere.^8^ Effect size estimates and local false sign rate output by mashr were used as metrics of QTL magnitude and activity, respectively. A local false sign rate less than 0.05 was used as a threshold for significant QTL activity.

### Annotation and Functional Enrichment Analysis

For the annotation of genomic regions and classification of variants according to their functional consequence we used the ENSEMBL Variant Effect Predictor (EMBL-EBI, Hinxton, UK).^61^ We used the *--pick* flag to extract a single annotation per variant following an ordered set of criteria to prioritize annotations. For functional enrichment analysis in regulatory regions distributed across the genome (Supplementary Table 14), we compiled a list of publicly available regions relevant for colon tissue from different studies (ie, active enhancers,^46^ variant enhancer loci,^46^ open chromatin sites,^34^^,^^46^ superenhancers,^62^ and transcription factor binding sites^63^). Regions from multiple samples of the same assay type were joined. In addition, we downloaded RNA binding protein sites, including splicing factor binding sites, from CLIPdb (Tsinghua University, Beijing, China).^64^ We used GREGOR (University of Michigan, Ann Arbor, MI),^65^ which defines enrichment (fold change) as the ratio between the number of observed vs expected SNPs overlapping the regulatory regions. This approach accounts for the number of LD proxies, gene proximity, and MAF.

### Phenotype Heritability Enrichment and Colocalization Analyses

For the SNP-based heritability enrichment analysis (partitioned heritability analysis) of eSNPs/sSNPs among disease-/trait-associated loci, we applied linkage disequilibrium score regression using the software LD SCore (Broad Institute of MIT, Cambridge, MA)^66^ with baselineLD model. A list with the GWAS summary statistics used for this analysis and related information can be found in Supplementary Table 15. Total SNP heritability for the tested phenotypes was estimated in observed scale for continuous traits and in liability scale for binary traits, using LD score regression from a total of 1,217,312 SNPs with a MAF greater than 0.05 in HapMap phase 3 populations (NHGRI, Bethesda, MD).^66^ Under the null hypothesis of all SNPs contributing equally to the total SNP-based heritability, we would expect that the 1122 sSNPs and 11,427 eSNPs identified in this study explain approximately 0.09% and 0.94%, respectively, of estimated total SNP heritability. Population prevalence and lifetime risk in the case of CRC was curated from the literature. For colocalization we used the fastENLOC (University of Michigan)^35^ approach. We computed Z-score–derived posterior inclusion probabilities for GWAS summary statistics with TORUS (University of Michigan)^67^ and assigned LD blocks to each locus using the references defined elsewhere.^68^ We performed multi-SNP fine-mapping analysis of eQTLs with DAP-G (University of Michigan).^69^

### Web Application

The web-based visualization resource CoTrEx was developed with the RStudio platform Shiny (Boston, MA)^70^ using open-source software.

### Data Availability

The RNA-Seq and SNP data that support the findings of this study as well as the sample covariates are available from the European Genome-phenome Archive under accession number EGAS00001004891. Complete summary statistics (including all FastQTL nominal pass results) for all QTLs identified in this study are available from the Digital Repository of the University of Barcelona at http://hdl.handle.net/2445/172697. Top-QTLs per gene are available in Supplementary Tables 7, 9, 10, 11, and 13.
