# Correction

**DOI:** 10.1016/j.jcmgh.2023.11.003

**Published:** 2023-12-13

Díez-Obrero V, Dampier CH, Moratalla-Navarro F, et al. Genetic Effects on Transcriptome Profiles in Colon Epithelium Provide Functional Insights for Genetic Risk Loci. Cell Mol Gastroenterol Hepatol 2021; 12:181-197.

In the above article, there is an error with the Data Availability statement. The first sentence of the Data Availability paragraph should be as follows:

The RNA-Seq and SNP data that support the findings of this study as well as the sample covariates are available from dbGaP genotypes and phenotypes repository under accession number phs003338.v1.p1.

